# Assessing the Capability of Large Language Models in Naturopathy Consultation

**DOI:** 10.7759/cureus.59457

**Published:** 2024-05-01

**Authors:** Himel Mondal, Satyalakshmi Komarraju, Sathyanath D, Shrikanth Muralidharan

**Affiliations:** 1 Physiology, All India Institute of Medical Sciences, Deoghar, Jharkhand, IND; 2 Naturopathy and Yoga, National Institute of Naturopathy, Pune, IND; 3 Research, National Institute of Naturopathy, Pune, IND

**Keywords:** perplexity, claude, large language models, copilot, gemini, chatgpt, referral and consultation, yoga, natural language processing, naturopathy

## Abstract

Background

The rapid advancements in natural language processing have brought about the widespread use of large language models (LLMs) across various medical domains. However, their effectiveness in specialized fields, such as naturopathy, remains relatively unexplored.

Objective

The study aimed to assess the capability of freely available LLM chatbots in providing naturopathy consultations for various types of diseases and disorders.

Methods

Five free LLMs (viz., Gemini, Copilot, ChatGPT, Claude, and Perplexity) were used to converse with 20 clinical cases (simulation of real-world scenarios). Each case had the case details and questions pertinent to naturopathy. The responses were presented to three naturopathy doctors with > 5 years of practice. The answers were rated by them on a five-point Likert-like scale for language fluency, coherence, accuracy, and relevancy. The average of these four attributes is termed perfection in his study.

Results

The overall score of the LLMs were Gemini 3.81±0.23, Copilot 4.34±0.28, ChatGPT 4.43±0.2, Claude 3.8±0.26, and Perplexity 3.91±0.28 (ANOVA F [3.034, 57.64] = 33.47, P <0.0001. Together, they showed overall ~80% perfection in consultation. The average measure intraclass correlation coefficient among the LLMs for the overall score was 0.463 (95% CI = -0.028 to 0.76), P = 0.03.

Conclusion

Although the LLM chatbots could help in providing naturopathy and yoga treatment consultation with approximately an overall fair level of perfection, their solution to the user varies across different chatbots and there was very low reliability among them.

## Introduction

The integration of large language models (LLMs) into healthcare systems has sparked significant interest due to their potential to revolutionize patient care and medical assistance [1,2]. These advanced generative AI systems possess the ability to comprehend and generate human-like text. This enables them to answer queries and even offer personalized recommendations based on vast repositories of medical knowledge [3]. The models have been tested for various capabilities in medical education, diagnostics, and patient care [4-6].

Naturopathy and yoga have gained traction as complementary medicine practices that emphasize holistic approaches to health and well-being. These modalities focus on the body's innate ability to heal itself through natural remedies, lifestyle modifications, and mind-body interventions [7]. As these practices gain acceptance within mainstream healthcare, there arises a need to explore how LLMs can augment naturopathic consultation and support individuals seeking alternative or integrative approaches to healthcare.

No previous study has explored the capability of LLMls in naturopathy consultation. The study will help in the evaluation of the effectiveness of LLM chatbots in understanding and responding to queries related to natural health remedies and mind-body practices. The study result will help researchers determine the chatbot’s capability of providing personalized recommendations tailored to individual needs and preferences. The performance of LLMs in naturopathic consultation contributes to the broader understanding of how AI technologies can integrate with diverse healthcare modalities.

## Materials and methods

Type and setting

The study was a cross-sectional and observational study. It was conducted with 20 clinical cases prepared for this study only. No data of patients were used. These cases were used to converse with five popular LLM chatbots - Gemini, Copilot, ChatGPT, Claude, and Perplexity. The study was conducted from December 2023 to January 2024. The chatbot Gemini was previously known as Google Bard, and Copilot was known as Microsoft Bing Chat. As this study involved only data audit from chatbots and there were no human or animal research participants, the study does not require any ethics review.

Clinical case preparation

**Figure 1:**
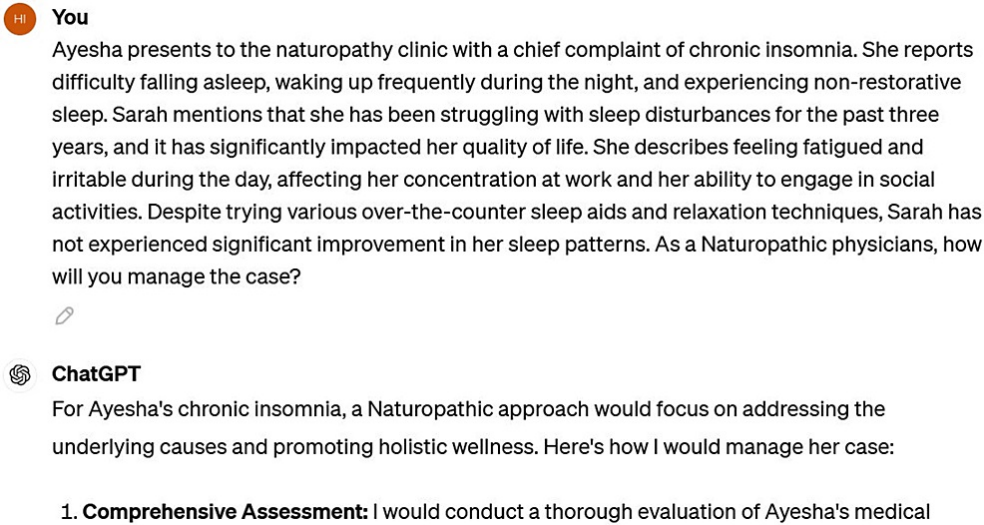
An example of a clinical case and part of the response of ChtGPT-3.5 GPT: Generative Pre-trained Transformer

Preparation of clinical cases involved the collaboration of five expert naturopathy doctors (with > 5 years of experience in patient care) who curated a diverse range of cases spanning various health conditions, treatment modalities, and patient profiles. A total of 20 such cases were created as a simulation of real-world clinical presentation. Each case was reviewed for content validity by another two experts with > 10 years of experience in naturopathy and yoga. An example of a case is shown in Figure 1.

Conversation with LLM

Two authors selected The LLM chatbots individually after reviewing previous studies and a consensus was reached to choose the final five. Conversations with five LLM chatbots, namely Gemini, Copilot, ChatGPT, Claude, and Perplexity, were conducted using the prepared clinical cases. The chatbots were tasked with responding to patient queries, providing information on naturopathic remedies, and offering recommendations for holistic health management. The interactions were structured to simulate authentic patient-provider dialogues, allowing for the evaluation of the chatbots' performance in understanding and addressing naturopathic concerns.

Scoring the responses

Three expert naturopathy physicians scored the responses based on predefined criteria, including language fluency, coherence, accuracy, and relevancy. The scoring guidelines are presented in Table 1.

**Table 1:** Guidelines for scoring large language model-generated contents Maximum achievable number 20, minimum 4, Average score was calculated from this data with the following formula: (Score of fluency + Coherence + Accuracy + Relevancy) / 4.

Parameter	Characteristic	Score
Fluency	Exceptionally fluent with precise and clear language	5
	Very fluent but with minor language issues	4
	Generally fluent, but occasional language concerns	3
	Noticeable language issues affecting comprehension	2
	Poor language fluency, making the content hard to understand	1
Coherence	Highly logical and well-structured	5
	Mostly logical, with minor coherence issues	4
	Generally logical, but some areas lack coherence	3
	Noticeable coherence issues impacting understanding	2
	Poor logical flow, making the content difficult to follow	1
Accuracy	Highly accurate, supported by robust scientific evidence	5
	Mostly accurate, with minor factual errors	4
	Generally accurate, but with notable inaccuracies	3
	Several factual errors impacting reliability	2
	Inaccurate information, undermining credibility	1
Relevancy	Highly relevant and applicable to the target audience	5
	Mostly relevant, with some areas needing improvement	4
	Generally relevant, but some content may not be useful	3
	Limited relevance, with substantial improvements needed	2
	Content lacks relevance and usefulness	1

Each response provided by the LLM chatbots was evaluated against these criteria to assess the quality and appropriateness of the information conveyed. Three doctors scored according to the guidelines and the average of the three was taken as the final score.

Statistical analysis

Data were presented as mean and standard deviation. The average score among three raters was taken as the final score for any category (e.g., for fluency of a chatbot, the score of three raters was added and divided by three). For an overall score of a chatbot for a particular case, all four attributes (fluency, coherence, accuracy, and relevancy score were added and divided by four). The final score of each LLM was also converted to percentage (score/4*100) and this percentage is termed perfection henceforth in this manuscript. The scores among five chatbots were compared by one-way analysis of variance (ANOVA). The agreement among the chatbots was tested by the intraclass correlation coefficient (ICC). We used GraphPad Prism 9.5.0 (GraphPad Software Inc., USA) for statistical analysis and considered a p-value <0.05 to be statistically significant.

## Results

**Figure 2:**
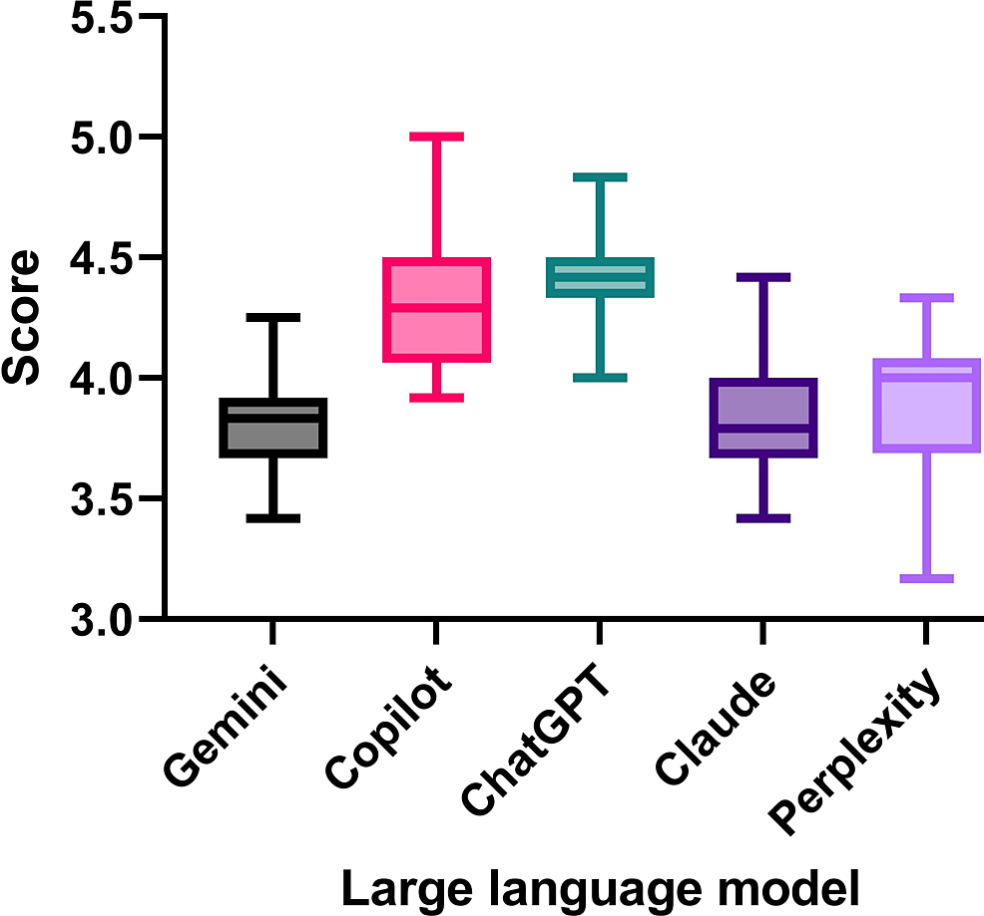
The average score of five large language model-based chatbots in naturopathy consultation GPT: Generative Pre-trained Transformer

The overall score of the LLMs were Gemini 3.81±0.23, Copilot 4.34±0.28, ChatGPT 4.43±0.2, Claude 3.8±0.26, and Perplexity 3.91±0.28 (ANOVA F [3.034, 57.64] = 33.47, p-value < 0.0001. The overall score is comparatively shown in Figure 2.

**Figure 3:**
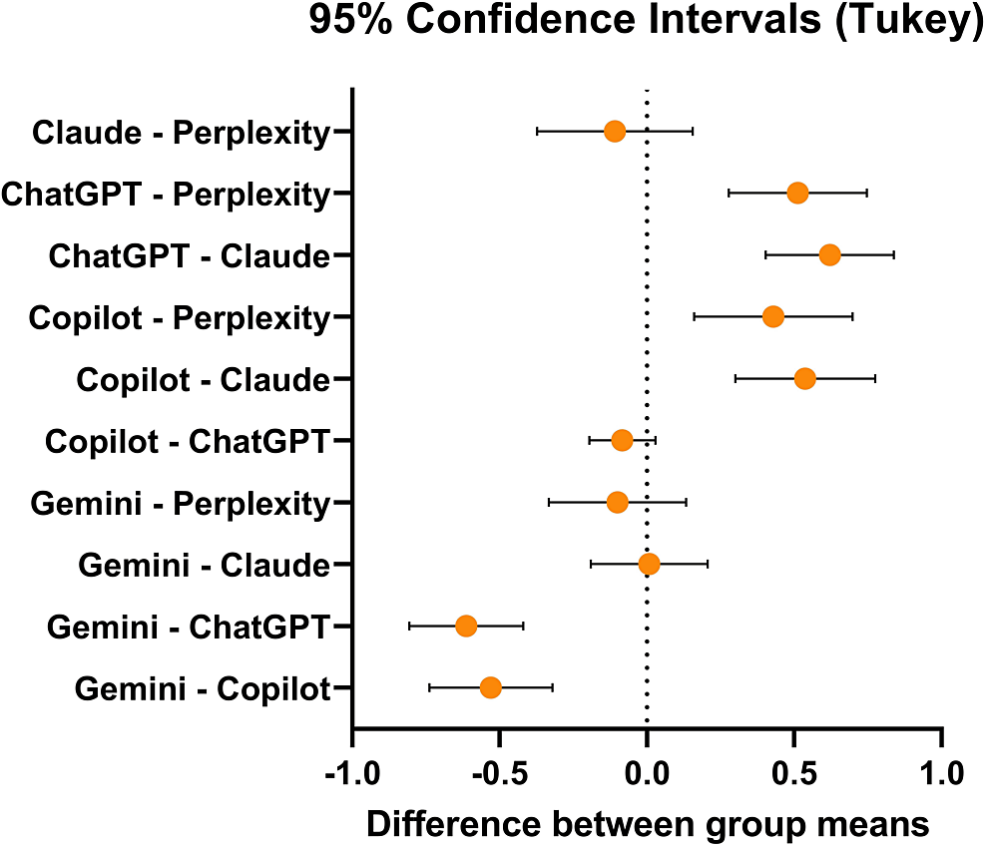
95% confidence interval of Tukey’s post-hoc test showing the relative difference between the scores of the groups

Among the chatbot, ChatGPT showed the highest score followed by Copilot. The post-hoc analysis is shown in Figure 3.

**Figure 4:**
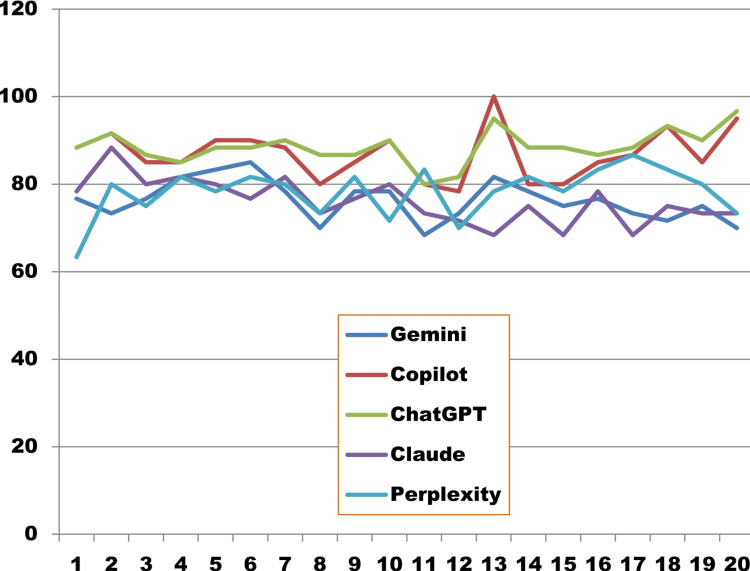
Case-wise percentage score of five large language models

The pair which do not intersect the midline showed a statistically significant score difference between them. Individual case-wise score (converted into percentages) is shown in Figure 4. Gemini got a perfection score of 76.25±4.55, Copilot 86.83±5.69, ChatGPT 88.5±3.93, Claude 76.08±5.14, and Perplexity 78.25±5.61.

The detailed scores in four domains are shown in Table 2.

**Table 2:** Domain-wise score of five chatbots Ge: Gemini, Co: Copilot, Ch: ChatGPT, Cl: Claude, Pe: Perplexity Tukey’s post-hoc test pairs indicate that those pairs had significant differences between their scores *Statistically significant p-value of analysis of variance (ANOVA)

Characteristics	Gemini	Copilot	ChatGPT	Claude	Perplexity	P-value	Tukey’s post-hoc significance
	Mean±SD						
Fluency	4.45±0.39	4.65±0.43	4.77±0.31	4.25±0.46	4.48±0.45	0.0003*	Ge-Co, Ge-Ch, Co-Cl, Ch-Cl
Coherence	4.53±0.4	4.65±0.48	4.78±0.35	4.45±0.38	4.38±0.51	0.0014*	Ge-Ch, Ch-Cl, Ch-Pe
Accuracy	3.28±0.45	4.28±0.61	4.35±0.44	3.42±0.57	3.53±0.67	<0.0001*	Ge-Co, Ge-Ch, Co-Cl, Co-Pe, Ch-Cl, Ch-Pe
Relevancy	2.98±0.55	3.78±0.76	3.8±0.63	3.1±0.29	3.25±0.58	0.0002*	Ge-Co, Ge-Ch, Co-Cl, Ch-Cl

All of them showed significant differences among them. The average measure ICC among the LLMs for the overall score was 0.463 (95% CI = -0.028 to 0.76), p-value = 0.03. This shows a poor reliability index (<0.5 ICC is considered poor).

## Discussion

The differences in overall scores among the LLMs - Gemini, Copilot, ChatGPT, Claude, and Perplexity suggest varying performance levels in naturopathy consultation. Some LLMs performed better than others in providing advice and guidance related to natural health remedies and practices. Hence, the response received by patients from those chatbots for naturopathy consultation help may get varying levels of response. Copilot and ChatGPT received the highest overall scores, indicating they performed better than the others. On the other hand, Gemini, Claude, and Perplexity scored lower, suggesting they may have struggled to provide accurate and relevant responses in the context of naturopathy. These differences in performance could be due to various factors such as the LLMs' training data and their understanding of naturopathic principles. It's important to continue refining LLMs to ensure they can effectively support both clinicians and patients in holistic healthcare practices like naturopathy.

A study from the United Kingdom found that ChatGPT and Gemini (previously known as Google Bard) were able to generate clinic letters and Google Bard showed lower compliance [8]. Researchers from India identified that the LLM may be used to perform the role of a teaching assistant and aid cosmetic surgery trainees in particular [9]. A systemic review found that an LLM-ChatGPT can help us with patient inquiries, note-taking, decision-making, trial enrollment, data management, decision assistance, research support, and patient education [10]. We in this study found a fair level of performance of LLMs in patient consultation. However, the current models might not be trained to its fullest capacity. That’s why many researchers are customizing their chatbots [11]. Hence, for Chinese traditional medicine, some researchers fine-tuned their model [12].

The agreement or consistency between the LLMs in their overall scores was not strong. One underlying reason for this poor reliability could be the inherent variability in how each LLM processes and generates responses. Factors such as the complexity of naturopathic principles, the diversity of patient cases, and the subjective nature of evaluating responses can contribute to discrepancies in scoring among the chatbots. If certain aspects of naturopathy were underrepresented or misrepresented in the training data, the chatbots may struggle to effectively address related queries or cases, further diminishing reliability. Previous studies also reported that the performance of different chatbots may vary. In multilevel testing, Bing Chat surpassed ChatGPT-3.5 and Google Bard for the Royal College of Ophthalmologists fellowship examinations [13]. ChatGPT had the greatest score, followed by Google Bard and Microsoft Bing in hematological case resolution [14]. Even for suggesting statistical tests, the performances of these models are different [15]. Along with that different versions of the models can also show differences in performance [16].

The study's findings have potential implications for the integration of LLMs in naturopathic consultation and holistic healthcare. Many of the patients who have access to devices and the internet can get a primary idea or second opinion about their disease from these freely available chatbots [17]. While some LLMs demonstrated promising capabilities in providing accurate and relevant guidance, the observed variability and poor reliability among chatbots underscore the need for further refinement and validation. LLMs need large data to be trained. This may be missing in Naturopathy due to the relatively smaller amount of literature in comparison to rich modern medicine literature.

This is the first study to test the capability of five LLMs in naturopathy consultation and their comparison in the consultation. The study result will help to understand the comparative perfection of chatbots in naturopathy consultation and accordingly patients can choose their suitable one. A limitation of the study is its reliance on simulated clinical cases and expert evaluations. These may not fully capture the complexities and nuances of real-world naturopathic consultations. Future research should aim to validate these findings in real-world clinical settings with diverse patient populations and explore additional factors that may impact the performance of LLMs in naturopathic consultation.

## Conclusions

While certain LLMs exhibited promising capabilities in providing accurate and relevant guidance on naturopathy and yoga consultation, variability and poor reliability among chatbots are evident. Harnessing the integration of LLMs in naturopathic consultation has the potential to enhance patient access to personalized, holistic healthcare solutions, thereby improving overall health outcomes and well-being. However, our study suggests that further improvement is required for using those LLMs in naturopathy and yoga consultation.
